# Signaling Pathways That Control Muscle Mass

**DOI:** 10.3390/ijms21134759

**Published:** 2020-07-04

**Authors:** Anna Vainshtein, Marco Sandri

**Affiliations:** 1 Craft Science Inc. Toronto, ON L4J 7S2, Canada; anna@craftscience.ca; 2 Veneto Institute of Molecular Medicine, via Orus 2, 35129 Padua, Italy; 3 Department of Biomedical Science, University of Padua, via G. Colombo 3, 35100 Padua, Italy; 4 Myology Center, University of Padua, via G. Colombo 3, 35100 Padova, Italy; 5 Department of Medicine, McGill University, Montreal, QC H3A 0G4, Canada

**Keywords:** skeletal muscle, atrophy, hypertrophy, sarcopenia

## Abstract

The loss of skeletal muscle mass under a wide range of acute and chronic maladies is associated with poor prognosis, reduced quality of life, and increased mortality. Decades of research indicate the importance of skeletal muscle for whole body metabolism, glucose homeostasis, as well as overall health and wellbeing. This tissue’s remarkable ability to rapidly and effectively adapt to changing environmental cues is a double-edged sword. Physiological adaptations that are beneficial throughout life become maladaptive during atrophic conditions. The atrophic program can be activated by mechanical, oxidative, and energetic distress, and is influenced by the availability of nutrients, growth factors, and cytokines. Largely governed by a transcription-dependent mechanism, this program impinges on multiple protein networks including various organelles as well as biosynthetic and quality control systems. Although modulating muscle function to prevent and treat disease is an enticing concept that has intrigued research teams for decades, a lack of thorough understanding of the molecular mechanisms and signaling pathways that control muscle mass, in addition to poor transferability of findings from rodents to humans, has obstructed efforts to develop effective treatments. Here, we review the progress made in unraveling the molecular mechanisms responsible for the regulation of muscle mass, as this continues to be an intensive area of research.

## 1. Introduction

In addition to its role in locomotion, skeletal muscle plays an essential role in whole body metabolism and glucose homeostasis, and serves as a protein reservoir [1]. This tissue’s remarkable ability to rapidly adapt to everchanging environmental and internal stimuli makes it vital for organismal health, survival, and adaptability. It is also clear that muscle is not static and has endo- and exocrine functions. Various myokines released from muscle act as messengers between skeletal muscle and other vital organs such as the liver, heart, fat, and even brain [2]. This signaling has consequences for certain chronic diseases such as diabetes, cardiovascular disease, cancer, cachexia, sarcopenia, and neurodegeneration, as well as many others. These relationships reaffirm the key and indispensable role of skeletal muscle in organismal health and survival. Indeed, many chronic diseases are accompanied and exacerbated by muscle wasting, and muscle metabolic state has been suggested as a disease modifier [1,3]. As the number of humans living with chronic diseases increases and the population continues to live longer, healthy aging and independent living requires the maintenance of muscle mass. It is therefore imperative, now more than ever before, to gain an intimate understanding of the molecular mechanisms that govern muscle mass and function.

In theory, muscle mass is determined through a simple balance between protein synthesis and protein degradation. During steady state conditions, protein synthesis equals protein degradation leading to proteostasis. Consistently, an increase in protein synthesis due to hormonal stimulation or resistance exercise leads to an increase in total muscle protein and organelles, resulting in increased muscle mass or hypertrophy. Whereas increased protein degradation due to reduced activity, defective neural input or increased proteolysis leads to muscle atrophy. However, the situation is more complicated, wherein the two seemingly opposing processes are typically active simultaneously, both during atrophy and hypertrophy. For instance, under catabolic conditions, protein breakdown by the proteasome and autophagy lysosome systems generate amino acids that can activate mechanistic target of rapamycin (mTOR) [4,5], the master regulator of protein synthesis. Moreover, induction of protein synthesis, by exercise for instance, also results in activation of protein breakdown as part of a cellular turnover program to rejuvenate the cell [6,7,8,9]. This creates a system of feedback loops that is more complicated than initially realized.

This is further complicated by the fact that skeletal muscle is not a homogenous tissue. Muscle is composed of a number of different fiber types that have distinct metabolic and biochemical properties. For instance, while fast twitch fibers are more sensitive to nutrient deprivation, they are more resilient to immobilization than slow twitch fibers. Moreover, different atrophic programs are activated in the different fiber types in response to the same stimulus, and of course the type of stimulus also dictates the atrophic response of any given myofiber. Indeed, most human skeletal muscles consist of mixed fiber types, and the precise fiber type composition depends on a number of factors such as genetics, sex, physical activity, and many other factors. The ability to alter fiber type composition through lifestyle changes grants muscle remarkable plasticity. Therefore, the susceptibility of various fiber types to different atrophic stimuli and their molecular response may vary significantly. This makes it difficult to generalize conclusions when studying whole muscle homogenates or any single muscle fiber type. It is therefore important to consider and investigate the differential response of the various fiber types and segregate findings on the basis of myofiber characteristics.

The molecular mechanisms underlying muscle atrophy and hypertrophy have been under intense investigation for several decades, and some major progress has been made in the field. This review focuses on recent discoveries and advances in the molecular mechanisms involved in the regulation of muscle mass and plasticity. A comprehensive review of all bona fide regulators of muscle mass, which have been previously rigorously reviewed [10], is beyond the scope of this review.

## 2. Regulators of Protein Synthesis

### 2.1. mTOR

During development, skeletal muscle growth is thought to be largely mediated by pathways converging on the metabolic master regulator mTOR. mTOR is a serine threonine protein kinase found at the epicenter of cellular growth regulation. mTOR integrates metabolic, hormonal, and mechanical signaling to determine the metabolic state of many cells including skeletal muscle. mTOR is activated by an auspicious environment that is conducive to growth. Nutrients (such as amino acids), hormonal stimulation (insulin/insulin-like growth factor 1 (IGF-1)), and mechanical and neural signaling activate mTOR, while inflammation (cytokines) and cellular stressors such as unfolded proteins inhibit mTOR activity [11]. Inhibition of mTOR during development either by rapamycin treatment or genetic manipulation results in the obstruction of cellular growth [12].

**Figure 1:**
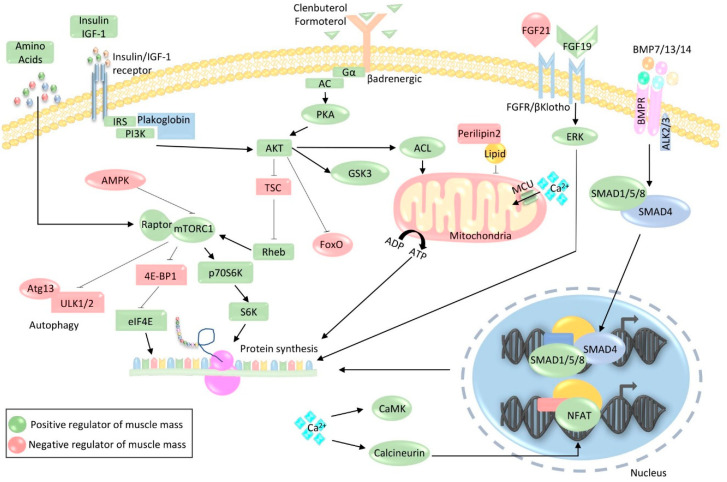
Pathways involved in protein synthesis. During muscle hypertrophy, the binding of insulin-like growth factor 1 (IGF-1)/insulin to their respective receptors activates IRS–PI3K–Akt–mTORC1 signaling, which is further stimulated by the presence of amino acids. The mTORC1 complex then enhances protein synthesis by inhibiting the eukaryotic translation initiator factor 4E (eIF4E) inhibitor 4E-binding protein 1 (4E-BP1) and activating p70S6K-S6-mediated protein translation. mTOR also blocks proteolysis by inhibiting autophagy initiation factors Unc-51 like autophagy activating kinase (ULK) 1/2. Clenbuterol and formoterol promote hypertrophy by binding to the adrenergic receptor and activating adenylate cyclase (AC)-PKA signaling, which culminates in the activation of Akt-mTOR. In addition to enhancing protein synthesis, Akt also blocks proteolysis by phosphorylating and inhibiting members of the forkhead box O family of transcription factors (FoxO), as well as activating glycogen synthase kinase (GSK) 3 and ATP citrate lyase (ACL). Fibroblast growth factor (FGF)19 enhances protein synthesis by binding to the βKlotho receptor and stimulating the extracellular signal-regulated kinase (ERK), which directly contributes to protein synthesis. Moreover, the binding of bone morphogenetic protein (BMP)7/13/14 to the BMPR receptor promotes muscle growth through the formation of a SMAD1/5/8–SMAD4 complex, which activates a hypertrophic transcriptional program. Calcium signaling also contributes to hypertrophy through the activation of calmodulin (CaM)-dependent kinase (CaMK) and phosphatase calcineurin, which stimulates the pro-growth transcription factor nuclear factor of activated T-cell (NFAT). Increased availability of nutrients and energy in the form of ATP further contribute to the auspicious environment that is permissive for muscle growth. Green shapes indicate positive regulators of muscle mass, whereas red shapes indicate negative regulators.

mTOR exists in two distinct complexes, the rapamycin-sensitive mTORC1 characterized by the presence of Raptor, and the rapamycin resistant mTORC2 characterized by the presence of Rictor. mTORC2 appears to regulate glucose and lipid homeostasis while mTORC1 regulates anabolic processes such as protein synthesis, as well as organelle biogenesis. mTORC1 regulates protein translation through enhancing both translational efficiency and capacity. mTOR enhances the cell’s translational efficiency by modulating p70S6K1, upstream of ribosomal protein S6 and factor 4E-binding protein 1 (4E-BP1), a negative regulator of ribosomal eukaryotic translation initiator factor 4E (eIF4E) [11] (Figure 1). mTOR has also been demonstrated to regulate cellular translational capacity by directly binding rDNA and enhancing ribosomal biogenesis [13]. In addition to activating translation, mTOR also inhibits muscle catabolism by blocking the cell’s main proteolytic pathways, the ubiquitin proteasome and the autophagy-lysosome systems [14]. True to its role, muscle-specific Rictor knockout mice do not display an overt phenotype, but exhibit decreased insulin-stimulated glucose uptake, as well as glucose intolerance [15]. A deficiency in Raptor, on the other hand, results in reduced postnatal growth, progressive dystrophy, impaired oxidative capacity, and increased glycogen stores. Moreover, pharmacological inhibition of mTORC1 blocks muscle hypertrophy in models of induced muscle growth such as post-natal development, synergist ablation, and muscle regeneration. However, although mTORC1 is necessary for mechanical load-induced hypertrophy, it does not appear to be required for a mechanical load-induced increase in protein synthesis [16]. Interestingly, the loss of mTOR’s catalytic activity seems to cause a more severe phenotype than the loss of mTOR protein. Conditional loss of mTOR concomitant with the expression of kinase-inactive mTOR results in early onset, rapidly progressing myopathy that impairs growth and leads to juvenile lethality. This was due to a more robust suppression of mTORC1 signaling, leading to more significant impairments in protein synthesis, oxidative metabolism, and autophagy. Stronger feedback activation of protein kinase B (PKB/Akt), and a dramatic downregulation of glycogen phosphorylase expression was also observed in this model, resulting in features reminiscent of glycogen storage disease type V [17]. Consistently, mTOR dysregulation has been documented in Pompe disease, a severe muscle wasting condition, caused by excessive accumulation of lysosomal glycogen. Reactivation of mTOR in the muscle of Pompe mice by tuberous sclerosis 1 (TSC1) knockdown, Ras homolog enriched in brain (Rheb) overexpression, or arginine resulted in the reversal of atrophy and a striking removal of autophagic buildup [18]. This is promising as a similar effect was observed in valosin-containing protein-inclusion body myopathy (VCP-IBM) muscle, where activation of mTOR led to amelioration of muscle atrophy [19]. Moreover, increased myofiber size was observed in murine muscle following transient expression of Rheb [20]. Therefore, lack of mTOR appears to result in myofiber degeneration and muscle myopathy, suggesting that mTOR is important for muscle growth, development, and survival. Another interesting observation was that the loss of mTOR signaling in these models did not result in excessive autophagy, despite its important inhibitory role in this process. Surprisingly, autophagy was impaired in these mice, which contributed to pathogenesis [12,17].

Interestingly, excessive mTOR activation appears to be equally detrimental. Genetic deletion of the mTOR inhibitor TSC1 in mice (which causes hyper activation of mTOR) results in a late-onset myopathy, which is related to impaired autophagy [21]. In these mice, constitutive and starvation-induced autophagy is blocked at the induction steps via mTORC1-mediated inhibition of ULK1, despite the activation of FoxO3. Rapamycin treatment or raptor depletion was sufficient to restore autophagy in these mice and improve the muscle phenotype [22]. Moreover, mTOR activation was found to contribute to denervation- and immobilization-induced atrophy [4,23], by both decreasing global protein synthesis through an increase in eIF4F complex formation [23] and inducing a FoxO-mediated catabolic program by blocking Akt [4]. Interestingly, rapamycin treatment was found to be sufficient to restore Akt activity and inhibit eIF4F complex formation, thereby mitigating muscle atrophy in both models. On the other hand, activation of mTOR by isometric contraction or through overexpression of Rheb enhanced mTOR signaling and rescued the immobilization-induced drop in protein synthesis and muscle atrophy [23].

A mounting body of evidence implicates the chronic activation of mTOR in aging skeletal muscle, where mTORC1 signaling is hyperactivated in muscle of sarcopenic mice and humans [24,25]. Chronic activation of mTOR in TSC1 knockout muscle results in mitochondrial dysfunction and increased oxidative stress, which over time ultimately leads to fiber decay. This appears to be at least in part due to increased expression of growth and differentiation factor 15 (GDF15) by Stat3 [24]. However, this effect could also be due to mTOR’s role in blocking autophagy, which is closely associated with muscle senescence [26]. Inhibition of mTORC1 in aging mouse reverses the senescent phenotype, resulting in the sparing of muscle mass [24,25].

Therefore, although mTOR is an important modulator of muscle growth during development, chronic activation of mTOR later in life appears to have the opposite effect. The precise mechanisms involved in this metabolic role reversal are not clear, and the exact point in time in which mTOR transforms from friend to foe, as well as the reason for this switch, remains a mystery. What is evident is that mTOR is entangled in an increasingly complex molecular web, which it may itself be weaving. It is therefore important to keep dissecting mTOR’s web and its role in the regulation of muscle mass.

### 2.2. Insulin/IGF-1

Upstream of mTOR, insulin, and insulin-like growth factor 1 (IGF-1) are well recognized for their role in muscle growth. IGF-1 and insulin act by binding to their respective receptors (Figure 1), which triggers the activation of several downstream kinases, culminating in the activation of Akt. Akt then mediates cellular growth by both enhancing protein synthesis, through the inhibition of GSK-3β and activation of mTOR, as well as blocking protein breakdown, through inhibition of the FoxO family of transcription factors. GSK-3β impairs protein synthesis by inhibiting eIF2Bε, and promotes atrophy through increasing the expression of atrophic E3 ligases, muscle ring finger1 (MuRF1) and Atrogin-1, as well as impairs muscle oxidative capacity by inhibiting mitochondrial biogenesis [27,28,29]. Blockade of GSK-3β suppresses muscle atrophy [27], facilitates a faster recovery from disuse atrophy [28], and improves muscle oxidative capacity [29]. The FoxO family controls skeletal muscle’s atrophy program by regulating the transcription of factors pertaining to both cellular proteolytic systems—the ubiquitin proteasome and autophagy lysosome. Thus, during atrophic conditions, reduced signaling through the IGF-1–Akt–mTOR axis contributes to atrophy not only through reduced protein synthesis, but also through increased degradation by releasing FoxO inhibition [30,31,32]. Moreover, reduced mTORC1 activity can also result in the activation of autophagy, as mTORC1-mediated phosphorylation inhibits the autophagy induction complex composed of Atg13 and ULK1 [30,33,34].

Although IGF-1 is synthesized predominantly in the liver, in response to growth hormone stimulation, recent evidence suggests that IGF-1 can also be produced by other peripheral tissues, including skeletal muscle [35]. In fact, overexpression of a muscle-specific isoform of IGF-1 strongly induces muscle growth and regeneration. Among the various isoforms, which differ mainly in their N-terminal signaling peptide and C-terminal extension peptide, IGF-1Ea appears to be the most effective in enhancing muscle growth and force generating capacity in young and aged mice [36].

While the importance of IGF-1 and insulin for muscle proteostasis and hypertrophy has been known for a long time, the relative roles of each remained elusive. In an attempt to tease this out, a recent study examined mice with muscle-specific deletions of the insulin receptor (IR), the IGF-1 receptor, or both. IR signaling contributed more to proteaostasis, and mice lacking this receptor had a marked reduction in muscle mass, whereas mice lacking IGF-1R did not. Nonetheless, the overlap of IR and IGF-1R signaling is critical to the regulation of muscle protein turnover as mice lacking both receptors displayed a marked reduction in muscle mass that was linked to increased proteolysis. Deletion of FoxO1, FoxO3, and FoxO4 in IR/IGF-1R double knockout mice reversed the increase in autophagy and completely rescued muscle mass without impacting proteasomal activity. Thus, insulin and IGF-1 work to suppress FoxO-regulated and autophagy-mediated protein degradation [37].

In addition to insulin and IGF-1, the desmosomal component plakoglobin was also found to bind the insulin receptor and p85 subunit of PI3K, with this interaction promoting PI3K–Akt–FOXO signaling. PI3K–p85 interaction is disrupted during atrophy by ubiquitin ligase tripartite motif-containing protein 32 (Trim32). Plakoglobin overexpression or inhibition of Trim32 was sufficient to increase PI3K–Akt–FOXO signaling, enhance glucose uptake, and induce fiber growth, whereas downregulation of Plakoglobin or overexpression of Trim32 had the opposite effect [38].

### 2.3. β2-Adrenergic Signaling

The beta-2 adrenergic receptor (β2 adrenoreceptor) is activated by epinephrine (adrenaline) and mediates the anabolic effects of β2-adrenergic agonists such as clenbuterol or formoterol (Figure 1). This effect is facilitated by Akt/mTOR signaling as treatment with rapamycin abolishes this effect [39]. Recent studies have demonstrated that β2-adrenergic signaling impinges on insulin/IGF-1 receptor signaling [40], where genetic or pharmacological inhibition of the insulin or IGF-1 receptor abolishes the anti-proteolytic effect of β2-adrenergic agonists. Interestingly, formoterol also induces the expression of JunB, a transcription factor that promotes muscle hypertrophy independently of mTOR [41].

### 2.4. Fibroblast Growth Factors (FGFs)

Fibroblast growth factors regulate a broad spectrum of biological functions, including cellular proliferation, survival, migration, and differentiation. They have been implicated in the regulation of self-renewal in skeletal muscle stem cells (satellite cells), as well as the maintenance and repair of skeletal muscle [42]. The signaling pathways downstream of FGFs include RAS/MAP kinase, PI3K/Akt, and PLC***γ*** pathways. Until recently, only two FGFs, FGF2 and FGF6, have been documented to influence muscle mass by regulating satellite cell function. Recently, FGF19 was found to positively regulate muscle mass during atrophy induced by glucocorticoid treatment, obesity, and sarcopenia [43]. FGF19 treatment was able to induce muscle hypertrophy, both in mice and human myotubes, through extracellular signal-regulated protein kinases 1 and 2 (ERK1/2) and S6K1-mediated signaling (Figure 1). This effect required the FGF19 receptor β-Klotho (KLB). Interestingly, the closely related family member FGF21 did not have the same effect [43]. On the contrary, FGF21 is released by atrophic muscle and mediates muscle atrophy and weakness by enhancing autophagy and mitophagy flux through stimulation of Bnip3 [44]. Mice lacking FGF21 in muscle did not experience loss of muscle mass or strength in response to fasting, whereas overexpression of FGF21 was sufficient to induce autophagy and muscle atrophy in mice. These contradictory findings among FGF family members are surprising since both FGF19 and FGF21 act on the same β-Klotho receptor [44]. Moreover, a recent study found that mitochondrial respiratory chain deficiency elicited a compensatory response in skeletal muscle by increasing the expression of FGF21, which resulted in enhanced mitochondrial function through an mTOR–YY1–PGC-1α-dependent mechanism [45]. Therefore, further research into FGFs, their target cell populations, and mechanisms is warranted.

### 2.5. Ions

Various ions are known to regulate muscle trophism. For instance, calcium signaling and dynamics are essential for muscle function and proteostasis. Intracellular calcium concentrations play a pivotal role in muscle physiology and either too much or too little can be detrimental. Calcium levels regulate calmodulin (CaM), which activates calcium/CaM-dependent kinases (CaMK) and phosphatases such as calcineurin (Cn), which stimulates the activity of nuclear factor of activated T-cell (NFAT; Figure 1). NFAT is a transcription factor involved in regulating muscle oxidative capacity as well as fiber size. This pathway was recently found to be hyperactivated in Myotonic Dystrophy type 1 (DM1) as a beneficial compensatory adaptation to the disease [46].

Altered zinc homeostasis also appears to play a role in the regulation of muscle mass. Metal-ion transporter ZRT- and IRT-like protein 14 (ZIP14) were shown to mediate cancer-induced cachexia. ZIP14-mediated zinc uptake into muscle progenitor cells represses the expression of MyoD and Mef2c, blocks muscle-cell differentiation, and induces loss of contractile proteins. Furthermore, ZIP14 is upregulated in cachectic muscle of mice and patients and can be induced by tumor necrosis factor α (TNFα) and transforming growth factor β (TGFβ). Deletion of ZIP14 markedly reduces cancer-induced muscle atrophy in mice [47]. Moreover, metallothionein 1 and 2, small proteins known to bind heavy metals such as zinc and copper, have also been implicated in the regulation of muscle mass. Simultaneous blockade of both metallothioneins results in the activation of Akt–mTOR signaling and myofiber growth, as well as spares muscle mass during glucocorticoid treatment. How these proteins activate Akt and protein synthesis is unclear, but gene expression analyses revealed an increase in IGF-1 and androgen receptor [48].

### 2.6. TGFβ

**Figure 2:**
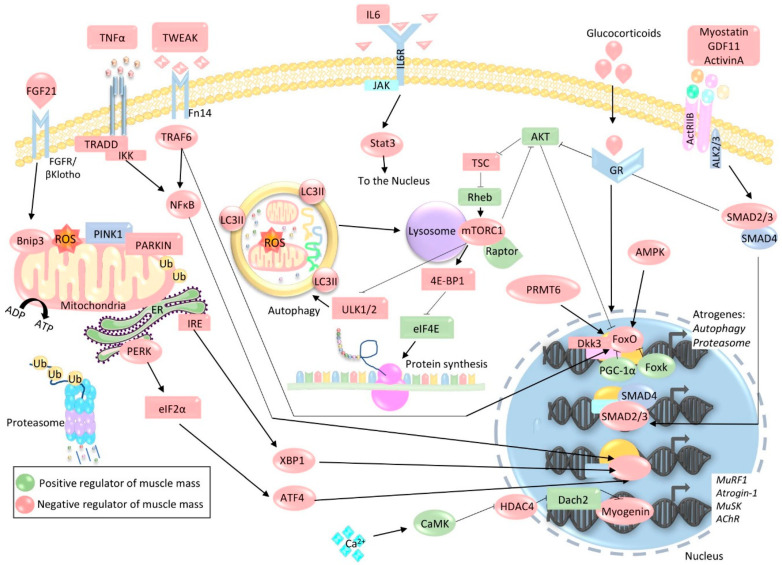
Pathways involved in protein degradation. During muscle atrophy, decreased binding of IGF-1/insulin to their respective receptors and/or increased binding of glucocorticoids to the glucocorticoid receptor (GR) result in reduced Akt/mTOR activation. This leads to diminished protein synthesis mediated by 4E-BP1-induced inhibition of eIF4E. Diminished mTOR activity also leads to the stimulation of autophagy through ULK1/2 signaling. At the same time, reduced Akt activity results in the release of FoxO from its cytosolic captivity, allowing it to activate an atrophic program by stimulating the expression of atrogenes belonging to the autophagy lysosome and ubiquitin proteasome proteolytic pathways. This sustains the increased proteolytic activity of these systems. The FoxO-induced atrophic program can be further enhanced by Dickkopf 3 (Dkk3) and blocked by PGC-1α and Foxk. Increased binding of activins (ActivinA, growth and differentiation factor 11 (GDF11)) and myostatin to the Activin type 2 (ActRIIB) receptor can contribute to atrophy through the activation of activin receptor-like kinase (ALK)2/3 and the formation of the SMAD2/3-SMAD4 complex, which in addition to further inhibiting Akt, also induces the expression of proteins involved in atrophy. Muscle atrophy is often accompanied by inflammation, which contributes to proteolysis. Increased levels of circulating cytokines such as tumor necrosis factor α (TNFα), TNF-like weak inducer of apoptosis (TWEAK), and IL6 leads to the activation of TRAF6, TRADD, IKK, and JAK which, in turn, activate NF-κB, Stat3, and FoxO, thus enhancing the expression of atrogenes. Mitochondrial dysfunction is also often observed during muscle atrophy, which leads to increased oxidative stress and mitophagy. Oxidative stress stimulates the release of IGF21, which then feeds back onto muscle, enhancing the expression of mitophagy receptor Bnip3. Accumulation of PINK1 and localization of E3 ubiquitin ligase Parkin as well as Bnip3 on the mitochondrial membrane lead to the elimination of dysfunctional mitochondria by mitophagy. Muscle atrophy is also associated with ER stress, which activates the unfolded protein response (UPR). UPR-activated IRE and PERK-eIF2α then promote atrophic transcriptional programs regulated by X-box binding protein 1 (XBP1) and ATF4, respectively. HDAC4, which is normally supressed by CaMK, is upregulated during denervation, resulting in the inhibition of Dach2, a transcriptional repressor of myogenin. This culminates in increased myogenin-mediated transcription of synaptic factors MuSK and AChR, as well as atrogenes muscle ring finger1 (MuRF1) and Atrogin-1. Green shapes indicate positive regulators of muscle mass, whereas red shapes indicate negative regulators.

The transforming growth factor β (TGFβ) superfamily is composed of 30 secreted proteins that govern a wide variety of cellular processes. The TGFβ family can be subdivided into two ligand subfamilies: the TGFβ/activin subfamily and the bone morphogenetic protein (BMP) subfamily, which appear to have opposite roles in skeletal muscle (Figure 1 and Figure 2). Perhaps the most well-known TGFβ family member is myostatin (growth and differentiation factor 8, GDF8), a potent negative regulator of muscle growth [49,50]. The hyper-muscularity of mammals including mice, dogs, sheep, cattle, and humans devoid of myostatin has fascinated scientists and body building enthusiasts for decades [49,51]. However, its biology has proved to be quite complicated. Myostatin and its TGFβ/activin family members can both enhance muscle growth and induce atrophy, depending on the downstream signaling they activate. These factors bind to plasma membrane receptors activin type IIA and type IIB (ActRIIA/B), as well as TGFβ receptors (TGFβRII), and negatively regulate muscle mass by activating activin receptor-like kinase (ALK)-4, -7, and -5, which in turn phosphorylate SMAD2/3 and promote the formation of a heterotrimeric complex with SMAD4 [52,53,54]. SMAD 2/3 can inhibit the transcription factor JunB, which normally promotes muscle growth and inhibits atrophy by blocking FoxO3 [41,52]. However, when myostatin/Activin/GDF11 are blocked, this SMAD2/3–SMAD4 complex can promote muscle growth. Although it is not clear how these factors regulate muscle mass, some evidence suggests that they impinge on the Akt/mTOR axis. Indeed, rapamycin treatment or mTOR knockdown diminishes the hypertrophic effect of myostatin blockade [52,54,55]. Moreover, SMAD3 can increase the expression of Atrogin-1 and inhibit Akt/mTOR signaling by negatively regulating the expression of miR-29 and -486, which inhibit the phosphatase and tensin homolog (PTEN) [56,57]. This results in increased Akt activation, thus enhancing protein synthesis. Surprisingly, myostatin is dispensable during denervation-induced muscle atrophy. In this scenario, denervation results in a blockage of IGF-1 signaling, and in increased levels of SMAD2/3, as well as E3 ubiquitin ligases Atrogin-1 and MuRF1. Interestingly, while SMAD2/3-deficient muscle is resistant to denervation-induced muscle atrophy [58], SMAD4-null mice are slightly atrophic and weaker than wild type litter mates, and exhibit exacerbated atrophy following denervation [58].

In contrast, BMPs act to prevent muscle atrophy by activating SMAD1/5/8, repressing MUSA1, a novel E3 ligase downstream of TGFβ, and activating mTOR signaling [59,60,61]. Indeed, BMP signaling is increased in models of muscle growth, while RNA interference of MUSA1 is protective against denervation-induced atrophy. Moreover, blocking TGFβ signaling through the administration of a soluble receptor (ActRIIB) was able to rescue muscle mass under several atrophic models, reverse cancer cachexia, and, remarkably, delay mortality independently of tumor progression [62]. Moreover, SMAD6 and 7, which are inhibitor proteins, prevent receptor-mediated activation of SMAD1/5/8 and SMAD2/3, respectively [61,63]. Although inhibiting the phosphorylation of SMAD1/5 exacerbates denervation-induced muscle atrophy, increasing BMP-SMAD1/5 activity is protective against this model of muscle atrophy [61]. Moreover, blocking BMP signaling by overexpression of BMP antagonist Noggin reverts the hypertrophic phenotype of Myostatin knockout mice, promoting the idea of genetic epistasis between the Activin/Myostatin and BMP pathways in skeletal muscle. Consistently, follistatin-mediated hypertrophy not only blocks myostatin signaling but simultaneously stimulates SMAD1/5/8 activation and repression of ankyrin repeat and SOCs box protein 2 (Asb2), a negative regulator of muscle mass [59,61,64]. Therefore, it appears that SMAD2/3 and SMAD1/5/8 compete for SMAD4 with opposing effects on muscle mass. Muscle hypertrophy involves crosstalk between BMP/ALK2/SMAD1/5/8 and Akt/mTOR pathways, where inhibition of mTOR signaling prevents BMP-mediated hypertrophy of skeletal muscle.

Thus, decades of research continue to highlight to importance of Akt/mTOR pathways in the regulation of muscle growth, as many molecular and intercellular messengers impinge on this pathway. However, the unexpected findings that mTOR counterproductively induces muscle atrophy under various atrophic conditions, including aging, is disconcerting. Therefore, further research into mTOR is warranted. Equally important is the identification of factors that are mTOR-independent, or those that can circumvent mTOR in the regulation of muscle mass.

## 3. Regulators of Proteolysis

The two major proteolytic pathways within skeletal muscle, the ubiquitin–proteasome and autophagy–lysosome systems, are responsible for the degradation of most proteins and organelles. However, two additional systems, the calcium-dependent calpain, and caspase systems are also responsible for some restricted proteolysis [65,66,67]. During atrophy, all systems appear to be working in unison, like a well-oiled disassembling line, with each responsible for its own repertoire of proteins to degrade at specific times during the process (Figure 2). Moreover, since muscle atrophy is a dynamic and continuous process, it also requires continuous transcriptional input to fuel the proteolytic pathways.

### 3.1. Ubiquitin–Proteasome System

The ubiquitin–proteasome system (UPS) is responsible for degrading most individual proteins. Components of the UPS are amongst the most commonly upregulated genes in various models of muscle atrophy including diabetes, denervation, cancer cachexia, fasting, and renal failure [68]. Proteins destined for degradation are first tagged by the addition of polyubiquitin chains, a process that involves three distinct components—E1, E2, and E3. E3 enzymes catalyze the rate-limiting step in ubiquitin conjugation and are extremely substrate-specific, providing selectivity to the UPS. Once tagged, the protein is delivered to the proteasome for digestion. Interestingly, E3 ligases auto-ubiquitinate, which makes them more susceptible to proteasomal degradation, thus requiring continuous transcriptional replenishment. Two muscle-specific E3 ubiquitin ligases, muscle ring finger1 (MuRF1/Trim63) and muscle atrophy F-box (Atrogin-1/MAFbx), are dramatically upregulated during various types of atrophy [68,69,70,71]. Moreover, mice deficient in MuRF1 and Atrogin-1 are partially protected against disuse atrophy [69]. MuRF1 appears to tag muscle structural proteins for degradation, whereas Atrogin-1 targets proteins involved in growth and survival pathways. Atrogin-1 was also found to regulate the proteolysis of autophagy–lysosome-related proteins in cardiac tissue [72]. However, various catabolic conditions, which result in atrophy, elicit vastly different molecular responses within skeletal muscle. For instance, MuRF1 knockout animals are not protected from fasting or microgravity-induced atrophy, and are only partially protected from denervation-induced atrophy [69,73]. However, these mice are protected from glucocorticoid treatment and aging-related loss of muscle mass [74,75,76], although MuRF1-null aged muscle remains weaker [76]. Loss of Atrogin-1, on the other hand, does not protect from glucocorticoid-, denervation-, or aging-induced muscle wasting [59,74,76,77], and only partially protects from starvation-induced atrophy [78]. Atrogin-1 knockout mice actually die prematurely from hypertrophic cardiomyopathy [72,76].

Although MuRF1 and Atrogin-1 play a dominant role in muscle atrophy, over 650 E3 ubiquitin ligases have been identified within the genome, and additional E3 ligases involved in muscle atrophy continue to be recognized. For example, Trim32 is responsible for the degradation of thin filaments, desmin, and z-band components, and inhibition or downregulation of Trim32 during atrophic conditions is protective against muscle loss [79]. Interestingly, Trim32 also functions as an inhibitor of PI3K–Akt–FoxO-pro growth signaling by regulating plakoglobin–PI3K binding [38]. Moreover, Trim32 has been recently documented to induce autophagy during muscle atrophy by interacting with ULK1 and AMBRA1 [80]. Tumor necrosis factor receptor-associated factor 6 (TRAF6) is another E3 ligase that is upregulated during muscle atrophy, and acts upstream of NF-κB [81]. TRAF6 catalyzes the conjugation of ubiquitin to the Lys63 residue of its target proteins. Lys63-linked ubiquitination is more commonly associated with autophagic degradation because it is recognized by the adaptor protein p62, as compared to Lys48-linked polyubiquitin chains that signal proteasome-dependent degradation [82,83]. Indeed, TRAF6-dependent Lys63 polyubiquitination stabilizes and enhances ULK1 activity, which is permissive for autophagy induction [84]. Mice lacking TRAF6 are protected against atrophy induced by denervation, cancer, and starvation [81]. This protection involves not only a significant reduction in polyubiquitinated proteins, but also reduced induction of fellow E3 ligases Atrogin-1 and MuRF1. This may be because TRAF6 is required for the optimal activation of JNK, AMPK, FoxO3, and NF-κB [81]. Therefore, TRAF6 appears to coordinate both major proteolytic pathways in response to atrophic stimuli in skeletal muscle.

Additional novel E3 ubiquitin ligases belonging to the Skp1–cullin-1–FBOX E3 ubiquitin ligase complex (SCF) have been identified and found to contribute to muscle loss during fasting, denervation, and cancer cachexia [59,85,86,87]. MUSA1 (*Fbxo30*) [59,71], SMART (*Fbxo21*), and Fbxo31 [85] are all induced during atrophic conditions and their inhibition materially reduces muscle wasting. Moreover, a recent study found SMART and Fbxo31 to be upregulated in a rodent model of critical illness-induced myopathy, and this upregulation was blunted by passive mechanical loading [88]. Although these ubiquitin E3 ligases contribute to proteolysis under various atrophic stimuli, they are not, on their own, sufficient to induce atrophy, with the sole exception of Ankyrin repeat and SOCS box-containing 2b (Asb2b) [64]. This E3 ligase must be suppressed to induce muscle growth, and its overexpression alone results in atrophy. However, the substrates of this ligase remain unidentified and warrant further investigation.

To make matters even more complicated, the ubiquitination process is reversible, and targets can be de-ubiquitinated by ubiquitin-specific hydrolases. Thus far, only two such proteases, ubiquitin carboxyl-terminal hydrolase (USP)14 and USP19, have been found to be upregulated under atrophic conditions [70,89]. Interestingly, the expression of USP19 correlates with that of MuRF1 and Atrogin-1 in skeletal muscles from patients with lung or gastrointestinal cancer. Moreover, knockout or knockdown of USP19 in mice and muscle cells partially protects muscle from glucocorticoid- and denervation-induced atrophy [90,91]. Although this presents an enticing point of regulation, to date, little is known about these enzymes in skeletal muscle.

The upregulation in E3 ligases during atrophy must be accompanied by increased proteasomal capacity in order to accommodate the surge in demand for protein breakdown. Indeed, the expression of several proteasomal subunits is increased during atrophy. Proteasomal subunit PSMD13 is upregulated in atrophying muscle from both lung cancer and renal failure patients [71]. Consistently, zinc finger protein ZNF216 (also known as ZFAND5) was found to bind the 26S proteasome and enhance its proteolytic activity by increasing ATP hydrolysis [92]. Like many other proteolytic proteins, ZNF216 is regulated by FoxO transcription factors, and mice lacking ZNF2016 exhibit decreased denervation-induced atrophy [93]. Post-translational modification of proteasomal subunits also augments proteasomal activity under muscle-wasting conditions. For instance, in mice fasted for 12–48 h, the levels of cAMP, PKA-dependent Rpn6 phosphorylation, and proteasomal activities were all increased without changes in proteasomal protein content [94].

Although the role of the ubiquitin proteasome system in muscle proteolysis is well established, the role, if any, this system plays in muscle growth has only recently been investigated. In both *Drosophila* and mouse myofibers, loss of UBR4, the N-end rule ubiquitin-protein ligase, induces hypertrophy by reducing the ubiquitination and degradation of a core set of target proteins, including the HAT1/RBBP4/RBBP7 histone-binding complex [95]. Interestingly, the importance of N-end rule ubiquitination in the regulation of muscle proteolysis was recognized over two decades ago. Indeed, this proteolytic mechanism is responsible for up to 60% of ATP-dependent degradation of soluble proteins in extracts from normal skeletal muscles, but not other cell types. Additionally, the activity of the N-end rule pathway increases in physiological and pathological states where muscle protein breakdown is elevated. More research into this pathway and its role in the regulation of muscle mass is therefore strongly warranted. Interestingly, the de-ubiquitinating enzyme USP19 is also involved in the regulation of myofibrillar protein transcription [91]. Indeed, its depletion increases the transcript levels of the MHC tropomyosin in a myogenin-dependant way. These findings indicate that the ubiquitin system not only mediates the increased protein breakdown but is also involved in modulating protein synthesis in atrophying skeletal muscle.

Although the UPS greatly contributes to muscle atrophy by degrading a large mass of proteins, this system cannot degrade intact myofibrils or organelles and therefore cannot alone account for muscle atrophy [67]. This suggests that proteolytic systems work in unison to promote muscular atrophy.

### 3.2. The Autophagy-Lysosomal System

The autophagy–lysosome system allows for the bulk degradation of long-lived organelles and superfluous proteins. In this process, tagged portions of the cytoplasm, dysfunctional organelles, and protein aggregates are sequestered into double membrane vesicles. These vesicles are known as autophagosomes and are delivered to the lysosome for proteolysis. A mouse line expressing green fluorescent protein tagged microtubule-associated proteins 1A/1B light chain 3 (LC3; an essential factor responsible for autophagosome formation), revealed muscle to have one of highest rates of autophagy during nutrient depletion [96]. Interestingly, glycolytic type II muscles display a higher content of autophagosomes than slow oxidative type I muscles, and indeed autophagy flux is higher in these muscles, both basally and in response to fasting [96,97]. Moreover, hyperactivation of autophagy exacerbates muscle atrophy induced by a myriad of naturally occurring and experimentally manipulated conditions, including cancer cachexia, fasting, sepsis, critical illness, cirrhosis, chemotherapy, disuse, denervation, COPD, constitutively active FoxO3, and by oxidative stress resulting from a mutation in superoxide dismutase (SOD1^G93A^), a mouse model of amyotrophic lateral sclerosis (ALS). Reducing autophagy by LC3 knockdown preserves muscle mass under certain atrophic conditions [98].

However, a basal level autophagy is important for the degradation of damaged and superfluous cellular components, thus acting as a housekeeping mechanism and promoting continuous cellular turnover which is vital for stress resistance. Indeed, muscle-specific deficiencies in the essential autophagy factors Atg5 or Atg7, VPS15, ULK2, AMPK, and mTOR result in loss of force generating capacity, activation of unfolded protein response, accumulation of dysfunctional organelles and damaged proteins, culminating in myofiber degeneration [22,99,100,101,102,103]. Loss of autophagy also results in an exacerbation of muscle wasting in response to atrophic stimuli such as starvation and denervation. Moreover, deficient autophagy is implicated in a myriad of myopathies and muscular dystrophies [99,104,105,106,107], which can be rescued by the reactivation of this process [99,104,105,106]. Thus, autophagy is required for muscle mass maintenance, and is critical for the quality control of skeletal muscle organelles; however, over-active autophagy can result in excessive catabolism and atrophy.

Evidence implicating aberrant autophagy in aging-induced atrophy have been accumulating. For instance, aged rats demonstrate higher expression of autophagy proteins basally; however, they are less able to further upregulate autophagy in response to perturbations, indicating reduced plasticity [108]. The accumulation of undegraded cellular materials in the form of lipofuscin is also suggestive of impaired autophagy in aged animals [108]. Moreover, the characteristics of autophagy-deficient muscle closely mirror those of sarcopenic muscle. Muscles deficient in Atg7 or TSC2 are characterized by degeneration of the neuromuscular junction, atrophy, and the loss of force generating capacity, leading to a premature aging phenotype and even death [26,100,109,110]. Interestingly, this phenotype is partially prevented by antioxidants, indicating the importance of autophagy in the elimination of oxidized proteins and dysfunctional mitochondria [26,109,111]. Similarly, removal of dysfunctional mitochondria by stimulating mitophagy with urolithin A, prolongs lifespan in the roundworm, *Caenorhabditis elegans*, and improves muscle function is aged rodents [112]. Moreover, chronic caloric restriction, with or without exercise, was found to activate autophagy and improve muscle health with aging [113].

Therefore, aside from its catabolic role, autophagy is responsible for cellular quality control and proper organelle turnover which is vital for muscle health and homeostasis. However, autophagy must be kept in check, since, if gone rogue, it can result in excessive catabolism and muscle wasting.

### 3.3. VCP/P97

Skeletal muscle ultrastructure is composed of highly organized and tightly packed myofibrillar proteins. This presents a logistical problem for degradation pathways, where contractile elements and proteins found within large complexes are not easily accessible to large and bulky degradation systems. Thus, a mechanism to release, extract, and deliver these proteins for proteolysis must exist. The valosin-containing protein (VCP/p97) is an ATPase responsible for segregating ubiquitin-tagged proteins from large cellular structures such as protein assemblies, organelle membranes, and alike, and chaperoning them for degradation by the proteasome or lysosome. Consistently, VCP/p97 and its co-factors, Ufd1 and p47 were found to associate with specific myofibrillar proteins and accelerate protein degradation during muscle atrophy, via both the proteasomal and autophagic pathways [114]. This suggests a role for p97 in extracting ubiquitinated proteins from myofibrillar structures [114]. Interestingly, p97 is methylated by methyltransferase-like 21e (Mettl21e) and Meetl21c [115,116], and loss of this methylation reduces its ATPase activity, leading to impairments in proteasomal [115] and autophagosomal [116] protein degradation.

Therefore, the various proteolytic systems in skeletal muscle work in concert to promote protein breakdown, acting as de facto boots on the ground to mediate atrophy. Each system contributes, albeit to varying degrees, to proteolysis, thus inhibiting one system has not been sufficient to fully block atrophy, as the others tend to compensate and pick up the slack.

## 4. Transcriptional Regulators of Muscle Mass

Although the transcriptional regulation of atrophy is complex and largely stimulus-dependent, comparative gene expression profiling of a number of atrophy models resulted in the discovery of several genes that are coordinately regulated under the various atrophic conditions [68,69]. These genes are known collectively as atrophy-related genes (atrogenes) and include molecules involved in both the ubiquitin–proteasome and the autophagy–lysosome proteolytic systems. Interestingly, several cellular signaling pathways have been documented to converge on the transcription of these key atrophic components. The FoxO family of transcription factors [117,118,119], NF-κB [120,121], the HDAC4-myogenin axis, and factors downstream of TGFβ [49,59,60] have all been demonstrated to impinge on the atrophic transcriptional program, culminating in the induction of atrogenes (Figure 2).

### 4.1. FoxOs

The FoxO family of transcriptional regulators consists of FoxO1, 3, 4, and 6, and is involved in a wide range of biological processes including metabolism, cellular proliferation and differentiation, apoptosis, longevity, and muscle mass regulation [122,123]. In skeletal muscle, FoxO1 and FoxO3 have both been documented to induce muscle atrophy in response to disuse and metabolic distress [68,118,119]. Overexpression of FoxO3 alone is sufficient to induce muscle atrophy [117,118,119] through the upregulation of E3 ligases [118], as well as the autophagy factors LC3 and BNIP3 [117,119]. Moreover, knockdown of FoxO3, or the expression of dominant negative FoxO3, blocks the induction of Atrogin-1 and MuRF1 under some atrophic conditions [118,124,125]. Similar to FoxO3, FoxO1 can also induce muscle atrophy by upregulating Atrogin-1 and MuRF1 [122,124], and the inhibition or genetic deletion of multiple FoxOs completely prevents the loss of muscle mass and force generating capacity in response to various atrophic stimuli, including fasting, glucocorticoid treatment, diabetes, limb immobilization, as well as partially spare muscle during denervation-induced atrophy [4,85,126,127].

FoxO activity is all about location, and these transcription factors must be de-phosphorylated before they are allowed entry into the nucleus. During atrophy, decreased levels of Akt leave FoxOs uninhibited and free to enter the nucleus. Moreover, AMPK [128,129,130], GSK1 [131,132], p38 MAPK [133], ERK [133,134], JNK [135,136], IKK [137], and MST1 [138,139] all phosphorylate FoxOs and regulate their activity. In addition to phosphorylation, the activity of FoxO transcription factors can be regulated by other posttranslational modifications, including acetylation, ubiquitination, and methylation [140]. For instance, acetylation of FoxO3 by p300/CBP inhibits FoxO3-mediated muscle atrophy [130,141], whereas deacetylation by HDAC1 activates FoxO and is both sufficient and required to induce muscle atrophy [141]. Surprisingly, deacetylation by Sirt1 has the opposite effect, inhibiting FoxO1 and 3, thereby blocking muscle atrophy and promoting growth [142]. Moreover, methylation by the arginine methyl transferase PRMT6 activates FoxO3. PRMT1, in turn, negatively regulates PRMT6 where PRMT1-deficient muscles exhibit hyperactive FoxO3 and muscle atrophy [143]. The capacity of FoxO3 to be post-translationally modified allows for the rapid potentiation of the atrophy program, which also presents an attractive target for pharmacological manipulation.

Interestingly, PGC-1α and β can inhibit FoxO3 activity on the promoters of atrogenes, thus rescuing muscle mass during denervation and fasting [144,145]. Glucocorticoid receptor and β-catenin, on the other hand, work synergistically with FoxOs to activate the transcription of atrogenes and induce muscle atrophy [146,147]. Moreover, secreted glycoprotein Dickkopf 3 (Dkk3) has been recently implicated in promoting aging sarcopenia by enhancing the nuclear import of β-catenin and promoting its interaction with FoxO3 [148].

FoxO transcription factors are also themselves transcriptionally regulated during catabolic conditions. Indeed, glucocorticoid receptor activation can directly induce FoxO mRNA expression [149,150,151]. Moreover, FoxO activity may also be indirectly impacted by chromatin remodeling. The bromodomain and extra-terminal domain (BET) protein BRD4 was found to be an epigenetic regulator of muscle mass. BET proteins appear to directly activate the muscle atrophy program during cachexia, and a pan-BET inhibitor protects tumor-bearing mice from loss of muscle and body mass, which dramatically prolongs survival, independent of tumor growth. In addition to their direct epigenetic action, BET proteins also coordinate an IL6-dependent AMPK nuclear signaling pathway converging on FoxO3, which contributes to cancer cachexia [152].

FoxO activity also appears to be counter-balanced by Foxk proteins (Foxk1 and Foxk2), which are transcriptional repressors of autophagy in muscle cells and fibroblasts [153]. FoxOs’ autophagic and atrophic transcriptional targets overlap quite a bit with those of Foxk, suggesting these two transcriptional regulators may act as the yin and yang of the muscle atrophy program. During nutrient-rich conditions, mTOR promotes the nuclear localization of Foxk1/2, which recruits Sin3A–HDAC complexes, thereby blocking histone H4 acetylation and restricting the expression of autophagy genes.

Therefore, FoxO transcription factors enable the progression of muscle atrophy by potentiating a transcriptional atrophic program that continuously fuels proteolytic pathways.

### 4.2. NF-κB

Inflammation often accompanies muscle atrophy, and can in itself activate an atrophic program under the command of transcription factor NF-κB. Under basal conditions, NF-κB is sequestered in the cytoplasm by the IκB family of inhibitors. Levels of cytokines such as tumor necrosis factor α (TNFα) and IL6 are increased in circulation during inflammation. The binding of TNFα to its receptor facilitates the proteasomal degradation of IκB, resulting in the translocation of NF-κB to the nucleus where it induces the transcription of atrogenes. Blocking NF-κB action through the overexpression of a negative regulator of this system (inhibitor of NF-κβalpha, Iκβα) is sufficient to prevent disuse atrophy [121,154], whereas overexpression of activated IkappaB kinase beta (IKKβ) in skeletal muscle promotes potent muscle wasting mediated, at least in part, by the ubiquitin-ligase MuRF1 [120].

Moreover, the cytokine TNF-like weak inducer of apoptosis (TWEAK) and its receptor Fn14 are also upregulated during muscle disuse [155,156], and mice lacking TWEAK are resistant to denervation. TWEAK–Fn14 regulates TRAF6 action, which coordinates the degradation of proteins and organelles via the proteasome and autophagy systems by activating NF-κB, AMPK, and FoxO catabolic signaling pathways [81]. In addition, a gene profiling study identified that the long noncoding RNA Atrolnc-1 is highly induced in cachectic muscle, which enhances NF-κB activity and MuRF1 transcription. Atrolnc-1 expression increases progressively in fasting mice and mice with chronic diseases such as cancer and kidney disease. In muscle cells, overexpression of Atrolnc-1 enhances protein breakdown while its suppression reduces serum starvation-induced proteolysis and attenuates muscle wasting in mice with chronic kidney disease [157].

Stat3 is another transcription factor that is activated by pro-inflammatory cytokines TNFα, IL6, and IL1. IL6 induces Stat3 phosphorylation by JAK, which promotes its nuclear localization and target gene expression under various atrophic conditions including cancer cachexia and sepsis [158]. Moreover, Stat3 activation appears to be both sufficient and required for muscle wasting [86,158,159].

Thus, inflammatory cytokines can contribute, or altogether induce, muscle wasting through the activation of pro-atrophy transcription factors NF-κB and Stat3.

### 4.3. HDAC4–Myogenin Axis

HDAC4 is a class IIa histone deacetylase that translates neural activity into actionable gene transcription within skeletal muscle [160]. In response to denervation, HDAC4 is dramatically upregulated and translocates from the neuromuscular junction to the nucleus. There, HDAC4 regulates the activity of E-box-binding basic helix-loop-helix muscle-specific transcription factor known as myogenin through the transcriptional repressor Dach2 [161]. Myogenin controls the expression of genes important for neuromuscular synapse formation, such as MuSK and nAChRs, which tend to be re-expressed in denervated, and neuromuscular disease-inflicted muscle. The upregulation of HDAC4, its close relative HDAC5, and myogenin following denervation culminates in increased expression of atrogenes MuRF1 and Atrogin-1 [162]. On the other hand, loss of either myogenin or HDAC4/5 during denervation diminishes the expression of Atrogin-1 and MuRF1 and attenuates muscle atrophy induced by denervation [160,162]. Postnatal deletion of HDAC4 in mice is protective against denervation-induced atrophy. HDAC4 also mediates induction of growth arrest and DNA damage-inducible 45α (Gadd45a), a small myonuclear protein that is required for denervation-induced muscle atrophy. A recent study, which aimed at uncovering additional HDAC4 substrates in skeletal muscle, identified myosin heavy chain, PGC-1α, and heat shock cognate 71 kDa protein (Hsc70) to all be regulated by HDAC4 [163]. Thus, HDAC4 is an important regulator of muscle trophism in denervation and NMJ disorders, but more research is required to better understand its signaling network and mechanism of action within skeletal muscle.

### 4.4. UPR

The unfolded protein response (UPR) is a cellular stress response system that is activated when unfolded or misfolded proteins accumulate in the lumen of the ER, causing ER stress. The ultimate goal of this mechanism is to restore cellular homeostasis by reducing protein translation, increasing protein degradation, and boosting ER folding capacity. Evidence implicating this process in the regulation of muscle atrophy have been mounting, and various factors involved in the UPR have been documented to promote muscle atrophy. For instance, UPR-related transcription factor ATF4 is activated in response to decreased protein translation stemming from unfolded protein-induced recruitment of GPR78/Bip and activation of PERK, a kinase that phosphorylates the initiation factor eIF2α and reduces ribosome assembly and protein synthesis. ATF4 contributes to muscle atrophy by modulating growth arrest and DNA damage-inducible 45a (Gadd45a), among others, in response to fasting, immobilization, and denervation [164,165]. Gadd45a induces nuclear remodeling by repressing genes involved in anabolic signaling and energy production such as PGC-1α and Akt, and induces pro-atrophy genes including autophagy and caspase-related genes [166,167,168,169]. Interestingly, loss of PERK either in vitro or in vivo counterintuitively results in the upregulation of autophagy lysosome, calpain, and ubiquitin proteasome genes, culminating in muscle atrophy [170]. However, the mechanisms of this effect are unclear and are not likely related to its role in activating ATF4.

Another branch of the UPR has also recently been implicated in muscle wasting. Depletion of GRP/Bip due to unfolded proteins in the ER activates IRE, which induces the activity of transcription factor X-box binding protein 1 (XBP1), resulting in atrophy. Targeted ablation of XBP1 in muscle ameliorates muscle loss in tumor-bearing mice, while its overexpression promotes atrophy of cultured myotubes [171]. This study also demonstrated that XBP1 is activated by toll-like receptor (TLR) and myeloid differentiation primary response gene 88 (MyD88) signaling in tumor-bearing mice.

UPR pathways are activated in response to a myriad of catabolic conditions including models of mitochondrial dysfunction, which are characterized by muscle atrophy. For instance, acute deletion of mitochondrial fusion protein OPA1 or the fission protein DRP1 in skeletal muscle result not only in dysfunctional mitochondria, but also exhibit UPR activation [172,173] and loss of muscle mass, albeit by different mechanisms.

### 4.5. miRs

Finally, microRNAs (miRs) have also been documented to regulate muscle mass during disuse. MiRs are endogenous, noncoding, short (20–22 nucleotides) RNAs that are involved in a wide variety of cellular processes. These miRs recognize the 3′-untranslated regions of their target substrates and silence their expression by either inducing transcript degradation or by blocking translation. Atrophic conditions such as denervation, starvation, cancer cachexia, or aging result in altered miR expression profiles. For instance miR-206 and -21 are induced with denervation and are both sufficient and required for atrophy [174]. miR-182, on the other hand, targets FoxO3, thus attenuating atrophy-related gene expression [175]. Indeed, miR-182 levels are reduced in response to some atrophic stimuli. Additional miRs associated with muscle disuse include miR-1 and miR-133a, both of which are reduced with bed rest and denervation [176,177].

A recent study in pre- and post-natal swine suggested that TGFβ is a potential target of miR-21 and that mRNA expression levels of miR-21 and TGFβI are negatively correlated during muscle development. Moreover, miR-21 is involved in muscle development by regulating PI3K–Akt–mTOR signaling through TGFβI [178].

TNFα and IGF-1 have also been demonstrated to regulate miR expression during skeletal muscle differentiation in vitro, wherein miRs, in turn, reduced myoblast fusion capacity by targeting genes associated with axon guidance, MAPK signaling, focal adhesion, and neurotrophin signaling pathways. Inhibition of miR-155 in combination with overexpression of miR-503 partially abrogates the inhibitory effect of TNFα on myotube formation. Therefore, the inhibitory effects of TNFα or the growth promoting effects of IGF-1 on skeletal muscle differentiation include the deregulation of known muscle-regulatory miRs [179].

Altogether, it is evident that the transcriptional regulation of muscle atrophy is important for both induction and continuity of proteolysis. This regulation depends on the interplay between a wide range of pathways, which often converge on the transcription of common atrophic factors known as atrogenes.

## 5. Metabolic Regulators of Muscle Mass

Changes in muscle mass are often accompanied by alterations in cellular bioenergetics and metabolism, which can be both the cause and the effect. Indeed, protein synthesis accounts for almost 30% of cellular ATP consumption and therefore any alterations in protein synthesis have a strong influence and reliance on energy supply and demand within the cell. Moreover, alterations in fuel availability also effect cellular energetics and by that notion may impact protein synthesis. For instance, alterations in lipid composition due to depletion of Perilipin2, a protein involved in lipid storage, result in muscle hypertrophy and protection from denervation-induced atrophy [180]. This effect was independent of mTOR signaling.

Reductions in mitochondrial content and changes in organelle morphology are common among various types of atrophy, and atrophying muscles are characterized by both reduced mitochondrial biogenesis and increased mitochondrial elimination. For instance, 7 days of denervation result in a ~50% drop in mitochondrial density, which correlates closely with loss of muscle mass [181]. Interestingly, the decrease in mitochondrial content precedes atrophy, suggesting that organelle loss contributes to the decline in muscle mass. However, causality has not been directly determined.

The level of the mitochondrial master regulator PGC-1α and close family member PGC-1β plummet under various atrophic conditions including diabetes, renal failure, cancer cachexia, and denervation [144,182,183,184]. This drop closely correlates with diminished organelle biogenesis [183,184,185,186,187]. Moreover, overexpression of either PGC-1α or -β blocks protein degradation and spares muscle mass during a number of atrophic conditions [144,145]; however, this is not sufficient to induce protein synthesis or muscle hypertrophy on its own. Various spliced isoforms of PGC-1α have been identified as playing different roles in the regulation of muscle mass. PGC-1α isoform 4 (PGC-1α 4), for instance, can promote muscle growth by inducing IGF-1 and repressing myostatin signaling [188]. Moreover, PGC-1α 4 expression is preferentially enhanced during resistance exercise, and mice with transgenic expression of this isoform have increased muscle mass and strength, and are resistant to atrophic stimuli. The anabolic effects of PGC-1α4 are dependent on GPR56, and knockdown of GPR56 attenuates PGC-1α4-induced muscle hypertrophy in vitro. Consistently, forced expression of GPR56 resulted in myotube hypertrophy through increased expression of IGF-1 [189].

Muscle atrophy is also accompanied by alterations in mitochondrial morphology [108,190]. Indeed, reduced levels of fusion proteins MFN2 and OPA1 with denervation result in fragmented mitochondria [190]. Small punctate mitochondria are less efficient at ATP production, more prone to ROS generation, as well as apoptosis, and are therefore predisposed for elimination by autophagy, all of which add to atrophy. Consistently, genetic deletion of OPA1 in skeletal muscle results in oxidative stress, which initiates an inflammatory response and premature cellular senescence culminating in a FoxO-dependent atrophy program and premature death [172]. The ablation of fission protein DRP1, on the other hand, does not appear to affect lifespan, but impacts calcium homeostasis by disrupting mitochondria–ER tethering, which results in mitochondrial calcium overload and reduced muscle strength [173]. Moreover, the overexpression of fission protein Fis1 is sufficient to induce muscle atrophy [191], which can be reversed if the mitochondrial network is restored [191]. Interestingly, two wrongs appear to make a right where concomitant ablation of both OPA1 and DRP1 mitigates the effect of OPA1 inhibition, resulting in a less severe mixed phenotype [192]. These findings underline the importance of balanced mitochondrial fission and fusion to muscle health [172,193].

An increasing number of factors that impinge on mitochondrial function are emerging as mediators of muscle atrophy. For instance, cytokine FGF21 promotes muscle loss by increasing Bnip3-dependant mitophagy [44]. However, the mechanism behind this regulation remains unknown. Casein kinase 2 (CK2) is an enzyme responsible for the phosphorylation mitochondrial import channel translocase of outer mitochondrial membrane 22 (TOM22); this phosphorylation is mandatory for proper mitochondrial protein import. Deletion of CK2 in mouse muscle results in reduced strength, abnormal metabolism, and accumulation of PINK1. PINK1 is a mitochondrial health sensor that is typically imported into the mitochondria and degraded. Accumulation of PINK1 on the mitochondrial membrane induces mitophagy by recruiting the E3 ubiquitin ligase Parkin, which ubiquitinates mitochondrial membrane proteins, effectively tagging the organelle for degradation. In muscles lacking CK2, autophagy flux is impaired, resulting in accumulation of undegraded materials and myopathy [194].

ATP citrate lyase (ACL) is a cytosolic enzyme that catalyzes mitochondria-derived citrate into oxaloacetate and acetyl-CoA. This enzyme appears to at least in part mediate IGF-1-induced muscle hypertrophy by increasing cardiolipin, and optimizing the activity of mitochondrial complexes and supercomplex. Thus, ACL may be acting as the molecular liaison between IGF-1-induced anabolic signaling (increasing energetic demand) and the activation of energy-producing mechanisms needed to supply the required ATP (increasing energy supply) [195]. However, the role of ACL does not end there; it also moonlights in myogenesis by regulating MYOD [196]. Furthermore, pyruvate dehydrogenase kinase 4 (PDK4), an important metabolic regulator, is elevated in several atrophic conditions including cancer, starvation, diabetes, and sepsis. Overexpression of PDK4 in myotubes promotes myofiber atrophy and mitochondrial abnormalities, whereas its blockage rescues myotube size in cells exposed to tumor conditioned media [197].

Consistently, the mitochondrial calcium uniporter (MCU) also positively regulates muscle mass [198]. Findings from a recent study demonstrate that overexpression of MCU can rescue muscle from denervation-induced atrophy by modulating PGC-1α4 and IGF-1-Akt signaling [199].

Therefore, mitochondrial ATP production, morphology, as well as signaling all influence muscle metabolic state. This is not surprising, given the important role mitochondria play in energy provision and the importance of ATP to muscle growth.

## 6. The Muscle Microenvironment and Stem Cell Niche

The idea that muscle is heavily influenced by its milieu is not new, and factors ranging from nutrients and metabolites to ions and cytokines appear to play a major role in cellular signaling and decision-making processes. It has been known for quite some time that malignant cells release atrophic factors and cytokines that affect muscle mass leading to cachexia. Among the various cytokines released by cancer cells, myostatin and activins have the greatest impact on skeletal muscle mass. Both are members of the TGFβ superfamily and contribute to cachexia by engaging Activin type I (Alk) and type II (ActRIIB) receptors, culminating in the expression of atrogenes [60] and muscle wasting. Indeed, genetically or biochemically interfering with these factors or their downstream targets is protective of muscle mass. Recently, tumor-derived Act-A was demonstrated to induce the expression of Twist1, a transcription factor important for mesenchymal stem cell fate determination. Induction of Twist1, in turn, drives expression of MuRF1 and Atrogin-1, leading to muscle protein degradation [200]. Genetic or pharmacological inactivation of Twist1 was sufficient to reverse muscle cachexia and improve survival in several genetic mouse models of cancer cachexia.

Cancer cells release not only cytokines but also extracellular vesicles (EVs). A recent study found these vesicles to carry chaperone proteins HSP70 and HSP90, which upon arrival into muscle activate TLR4, thereby inducing catabolism and contributing to cachexia [201].

Muscle wasting and cachexia are not only the result of increased muscle breakdown, but also a decreased repair capacity by satellite cells. Some recent studies provide insights into how the muscle stem cell niche and microenvironment contribute to the regulation of muscle mass [202]. This concept is perhaps best illustrated in tumor-bearing mice and oncology patients, where muscle cachexia is associated with impaired differentiation and incorporation of both satellite and non-satellite muscle progenitors. Although these cells are activated with cachexia, they are unable to completely differentiate due to persistent expression of the self-renewal factor Pax7, which is mediated by NF-κB. Overexpression of Pax7 alone induces atrophy in normal muscle, while its blockade reverses muscle wasting in tumor-bearing mice by restoring satellite cell function [203]. Another cell population that has been documented to contribute to muscle wasting with denervation is the fibro-adipogenic progenitors (FAPs). Like other muscle progenitors, these cells are activated following muscle injury. FAPs progressively accumulate following denervation, and FAPs from denervated muscles exhibit functional properties and gene expression profiles that are distinct from those obtained from muscles regenerating following cardiotoxin injury. FAPs activated by denervation or other pathological conditions such as spinal muscular atrophy (SMA) or amyotrophic lateral sclerosis (ALS) exhibited increased Stat3–IL6 signaling, which promoted muscle atrophy and fibrosis. Inactivation of this pathway was sufficient to counteract muscle atrophy and fibrosis in these atrophic models [204].

## 7. Conclusions

Although substantial progress has been made in our understanding of the molecular mechanisms that control muscle mass, we are still far from having the whole picture. The lack of viable treatments for muscle wasting conditions both as primary and secondary morbidities are perhaps the strongest testament to this. The importance of healthy muscle to whole body homeostasis and the vigor of other organs during health and disease brings muscle research to the forefront. We therefore must continue to unravel the mechanisms underlying muscle atrophy in their entirety to reveal their complexity. Moreover, our most common mediums for studying muscle atrophy rely on rodent and cell culture models, the findings from which do not appear to translate well to humans, thus delaying therapeutic development. Indeed, many therapeutic targets that seem promising in the lab, despite many efforts, never make it to the bedside. More rigorous testing must therefore be performed on potential therapeutic targets, along with increased efforts for developing models that better mimic human muscle behavior.
